# Update on Calcium and Phosphorus Requirements of Preterm Infants and Recommendations for Enteral Mineral Intake

**DOI:** 10.3390/nu13051470

**Published:** 2021-04-27

**Authors:** Walter Mihatsch, Ulrich Thome, Miguel Saenz de Pipaon

**Affiliations:** 1 Department of Pediatrics, Ulm University, 89075 Ulm, Germany; 2 Department of Health Management, Neu-Ulm University of Applied Sciences, 89231 Neu-Ulm, Germany; 3 Division of Neonatology, Children’s Hospital, Department of Women’s and Children’s Health, University Hospital, 04103 Leipzig, Germany; Ulrich.Thome@medizin.uni-leipzig.de; 4 Department of Neonatology, Hospital Universitario La Paz, Department of Pediatrics, Universidad Autonoma de Madrid, 28046 Madrid, Spain; Miguel.saenz@salud.madrid.org

**Keywords:** preterm infant, bone mineral content, calcium requirements, phosphorus requirements, bone mineralization

## Abstract

Background: With current Ca and P recommendations for enteral nutrition, preterm infants, especially VLBW, fail to achieve a bone mineral content (BMC) equivalent to term infants. During the first 3 years, most notably in light at term equivalent age (<−2 Z score) VLBW infants’ BMC does not catch up. In adults born preterm with VLBW or SGA, lower adult bone mass, lower peak bone mass, and higher frequency of osteopenia/osteoporosis have been found, implying an increased risk for future bone fractures. The aim of the present narrative review was to provide recommendation for enteral mineral intake for improving bone mineral accretion. Methods: Current preterm infant mineral recommendations together with fetal and preterm infant physiology of mineral accretion were reviewed to provide recommendations for improving bone mineral accretion. Results: Current Ca and P recommendations systematically underestimate the needs, especially for Ca. Conclusion: Higher enteral fortifier/formula mineral content or individual supplementation is required. Higher general mineral intake (especially Ca) will most likely improve bone mineralization in preterm infants and possibly the long-term bone health. However, the nephrocalcinosis risk may increase in infants with high Ca absorption. Therefore, individual additional enteral Ca and/or P supplementations are recommended to improve current fortifier/formula mineral intake.

## 1. Introduction

Appropriate mineral and vitamin D intakes are essential for adequate bone health. The optimum target for mineral accretion in preterm infants on enteral nutrition is unknown. The widely accepted physiologically relevant outcome is an accretion of bone mass that is proportional to the linear growth weight gain as achieved by the fetus in utero and subsequently the term born infant over the first year [1,2,3,4,5].

### 1.1. Definition of Bone Mineral Deficiency of Prematurity

Bone mineral deficiency of prematurity (BMDP) defined as insufficient osteoid mineralization or reduced bone mineral content [6,7,8] caused by low or insufficient calcium and/or phosphate intake has been reviewed recently [9,10,11,12,13]. This condition is commonly referred to in the literature as “osteoporosis of prematurity”, “rickets of prematurity”, “osteomalacia of prematurity”, “osteopenia of prematurity”, or “metabolic bone disease of prematurity”.

### 1.2. Clinical Signs of BMDP in Preterm Infants

BMDP is associated with a variety of clinical signs, e.g., enamel hypoplasia and caries [14,15], fractures [16,17,18], radiological signs of diminished bone mineralization [9], myopia and against the rule astigmatism [19], and dolichocephalic head flattening together with deformation of the palate [20]. Incidences of BMDP of about 40% [9] in very low birthweight (VLBW) infants and 50% in extremely low birthweight (ELBW) infants [21] have been reported in the past. Fractures have been observed in about 30% of VLBW infants [22,23] in the eighties. In ELBW infants from that time, improved nutrition with enrichment with minerals have reduced the reported incidences of fractures at least down to 7–10% [24,25] and radiological BMDP down to 15% [26]. Even though the actual incidence of BMDP or fractures is unknown, ELBW infants are especially at risk [24,27]. Whereas dolichocephalic deformation of the skull is still seen in the clinical experience of the authors, fracturs are a rare event and only occasionally seen in extremely immature infants. Routine skeletal X-ray screening is therefore not recommended.

### 1.3. BMD in the First Years of Life

At term equivalent age, preterm infants grossly fail to meet the whole-body BMC of infants born at full term [28,29]. At 6 months of age, in a previous study, a similar BMC has been found in preterm infants as in a previous group of full term infants [28] and a catch-up of BMD in preterm infants within the first year of life has been hypothesized [30] and shown in a recent pilot study in which infants received extra mineral intake upon discharge until deemed appropriate [31]. In contrast, recent meticulously performed longitudinal data showed lower BMC, lower BMD and lower BMC/length^2^ in VLBW preterm infants throughout the first 3 years of life [32]. Particularly light for term equivalent age VLBW infants appear to be at highest risk.

### 1.4. BMD at Child Age and in Adults

For child age, conflicting data have been published. One study reports a clear trend towards a reduced BMD in former preterm infants, but no significant difference was found [33] whereas others report significantly reduced bone mineral density in former preterm infants when compared to term infants [34,35]. Previous reviews on bone health in adults did not indicate a significant effect of birth weight on adult BMC [36]. In the only available randomized trial, adult peak bone mass was not associated with early mineral intake [23]. However, “compared with population reference data, preterm subjects were significantly shorter and had lower lumbar spine bone mineral density; the deficits were greatest in those born small for gestational age” [23]. Several more recent observational studies in adults born preterm with VLBW or SGA found lower adult bone mass, lower peak bone mass (usually achieved by the 3rd decade of life, regarded as the most important determinant of osteoporosis) and higher frequency of osteopenia/osteoporosis, implying an increased future fracture risk [37,38,39,40,41,42]. The most pronounced bone deficits are seen in VLBW adults, suggesting a role of early life programming in skeletal development [37,39,41,42].

In conclusion, appropriate mineral (and vitamin D) intakes are essential for adequate bone health. The available data suggest that current enteral recommendations for Ca and P intake in preterm infants do not meet lifetime needs for all infants.

## 2. Materials and Methods

The present paper is a narrative review, with main focus: preterm infants in hospital up to 34 weeks of gestation because discharge programs will start thereafter. A PubMed search was performed to identify current recommendations for enteral nutrition of preterm infants published after the year 2000. All enteral recommendations were analyzed for their individual basis.

The physiology of fetal mineral accretion and preterm infants’ mineral absorption was reviewed because all enteral recommendations were based on these two pillars.

Finally, conclusions, enteral mineral recommendations, and future research recommendations were derived from the presented data.

## 3. Results

### 3.1. Current Recommendations for Enteral Ca and P Intake in Preterm Infants

Current enteral mineral intake recommendations are given in Table 1 [1,2,3,4,5]. Considerable differences among various experts’ recommendations were found. The lowest recommendation has been published by ESPGHAN in 2010 [2]. This recommendation is currently updated. The highest has been published by AAP in 2013 [3]. There is a remarkable range of recommended intakes of Ca 120–220 mg/kg/day and P 60–140 mg/kg/day. All recommendations advised to use the same intake for all preterm infants. No individualization of the intake has been recommended. There was a consensus in all recommendations that too low intake of Ca and P is associated with BMDP [1,2,3,4,5]. None of the recommendations provided data on side effects or toxicity of a higher Ca or P intake. None of the enteral recommendations was based on long term bone health.

### 3.2. Physiology of Ca and P Requirements

In all enteral recommendations, the estimations of the nutritional mineral requirements of the preterm infant have been based upon the combination of fetal mineral accretion combined with the range of mineral absorption rate by the preterm intestine [1,2,3,4,5]. The fetal mineral accretion during the third trimester was assumed to average mineral accretion rates of 90–120 mg/kg/day for Ca and 60–75 mg/kg/day for phosphorus [1,2,3,4,5]. However, estimation of fetal mineral accretion (factorial approach), based on measurements of fetal body composition and fetal weight gain, may be higher, as outlined below.

#### 3.2.1. Fetal Body Composition

**Figure 1:**
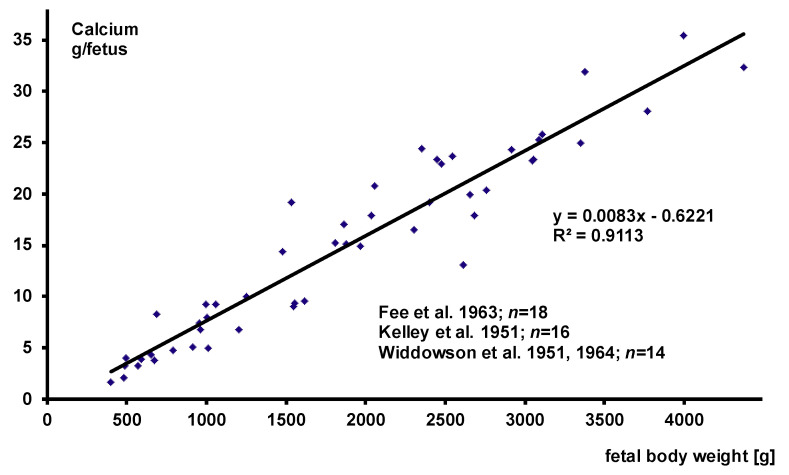
Fetal body composition—calcium.

**Figure 2:**
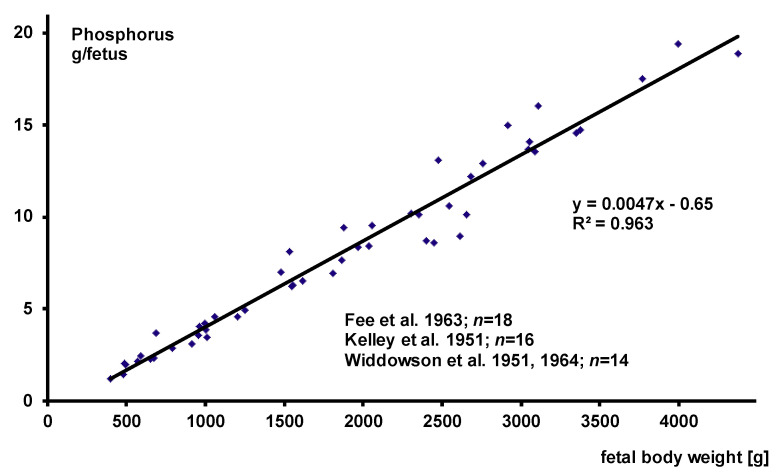
Fetal body composition—phosphorus.

Three major papers on body composition assessment of human fetuses at various stages of gestation have been published so far [43,44,45]. Figure 1 and Figure 2 give the calcium and phosphorus data of all analyzed fetuses in which total body analyses of P and Ca together have been performed. There is a strong linear association between fetal body weight and total calcium or total phosphorus measurement. Therefore, with regard to the Ca to P ratio, fetal body composition is constant throughout the analyzed weight range and absolute values depend on weight gain.

The data suggest that total body content of Ca and P increase by (8.3 mg) (0.21 mmol Ca) and 4.7 mg (0.15 mmol P) per gram of body weight. These data have been reconfirmed e.g., by delayed gamma neutron activation in preterm infants post mortem for Ca and P [46] and by dual energy x-ray absorptiometry (DXA) in stable term and preterm infants for Ca [47]. Both of these studies may slightly overestimate the steepness of the slope of the regression line, because infants were not analyzed right after birth and the smaller preterm infants are the more rapidly they demineralize after birth [46,47].

#### 3.2.2. Fetal Weight Gain

**Figure 3:**
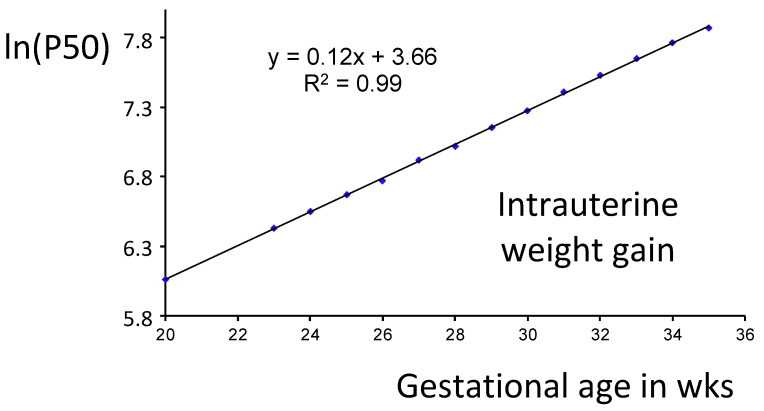
Natural logarithm of the 50th percentile of the German newborn infants’ growth reference.

Weight gain is obviously the major determinant of mineral requirements. The German newborn infant’s growth reference was based on the cross-sectional measurement of weight at birth of more than 1.8 million term and preterm infants, and is therefore regarded as one of the largest growth references available [48]. Figure 3 shows that there is a highly significant linear association between gestational age in weeks and the natural logarithms of the 50th weight percentile of this growth reference. These data suggest that the average fetal (intrauterine) weight gain of infants up to 34 weeks of gestation may mathematically be approximated by exponential weight gain of 17 g/kg/day.

On the basis of both cross-sectional BW data (basically the US growth reference by Alexander et al. [49]) and ultrasound fetal weight data, the recommendation by Klein concluded that intrauterine fetal weight gain is approximately 16–17 g/kg/day up to 34 weeks GA [5]. More recently, several additional fetal growth references have been analyzed by William Hay [50]. The average weight gain among this diverse population was 17 g/day/kg, ranging from 15 to 20 g/day/kg for average sized infants, slower for smaller infants and faster for larger infants. Therefore, recommendations may reasonably be based on the average fetal weight gain of 17 g/kg/day up to 34 weeks of gestation.

Given an average fetal weight gain of 17 g/kg/day the average fetal mineral accretion rate is 141 mg/kg/day (3.52 mmol/kg/day) for Ca and 80 mg/kg/day (2.58 mmol/kg/day) for phosphorus. These values are far above the assumptions on with the majority of recent recommendations were based on and provide a more appropriate average reference target. Approximately 98% of the calcium, 3.45 mmol/kg/day, is used for bone mineralization and deposited as microcrystalline apatite (Ca_5_(PO_4_)_3_) with a fixed molar Ca/P ratio of 1.67. The corresponding average phosphorus accretion in apatite is 2.07 mmol/kg/day. The remaining fetal phosphorus accretion is used for tissue accretion (0.5 mmol/kg/day). Average molar fetal whole-body Ca/P ratio is therefore 1.36. At slower weight gain (15 g/kg/day) the ideal average mineral accretion would be 125 mg/kg/day (3.1 mmol/kg/day) for Ca and 71 mg/kg/day (2.28 mmol/kg/day) for phosphorus. Faster target weight gain (20 g/kg/day) would increase the average mineral accretion target up to 164 mg/kg/day (4.09 mmol/kg/day) for Ca and 94 mg/kg/day (3.03 mmol/kg/day) for phosphorus.

#### 3.2.3. Ca and P Absorption

Average net intestinal Ca absorption in preterm infants (35–82%) may be slightly higher with multi-nutrient fortified human milk than with bovine milk based preterm formulas [1]. However, such observations are not consistent, because a variety of factors confound Ca absorption such as the source of the mineral salt and the amount and quality of fat, protein, carbohydrate and phosphorus in the diet [1]. In addition, the individual variability may be as high as 21% to 90% [1]. Basically, net calcium absorption from supplemented own mother’s milk or banked human milk or from preterm formulas fed to 1-month-old premature infants appears to be a linear function of intake at least in the range of 40–120 mg of calcium/(kg/day) [51]. In contrast, P absorption in preterm infants is usually at least 80% (reported range 60–95%) of the intake [1,4]. Therefore, it may be impossible to cover all individual infant’s needs, all formula and human milk supplement Ca/P bioavailability with only one given Ca and P concentration. The variations in Ca and P absorption explain most of the observed differences among various experts’ recommendations (Table 1). The neonatologist needs to know the Ca and P bioavailability of the various nutritional products used. This information should be provided by the manufacturers.

In some individual preterm infants, a net Ca and P retention closer to fetal accretion has been reported (e.g., see [1]). For instance, in a study providing 222 mg Ca/kg/day, Ca retention was 134 mg/kg/day [52]. However, given the average weight gain of 21 g/kg/day in this study, the achieved average mineral accretion rate was far below fetal mineral accretion (see Section 3.2.2.). On average, at term equivalent age and at 3 months of corrected age preterm infants still fail to achieve term infant whole-body bone mineral content (BMC) [28,29,32].

#### 3.2.4. Individualized Ca and P Supplementation

On the other hand, in individualized Ca and P supplementation (target: provision of a slight surplus defined by simultaneous urinary excretion of Ca and P in spot urine at low concentrations of 1–2 mmol/L) average fetal bone mineral accretion has been achieved and validated by single photon absorptiometry (SPA) and balance studies [53]. In these studies, considerably higher average Ca (260 mg/kg/day) intakes were required than recommended, whereas the average P intake (100 mg/kg/day) was within the recommended range (Table 1) [54]. Individualized enteral Ca/P supplementation has been studied successfully using organic Ca (Ca-gluconate) and P (Na_2_-glycerophosphate) salts (please see ref. [53,54] for details).

Additional Ca supplementation was required in the vast majority of infants on supplemented human milk feeding or preterm formula feeding. Gastrointestinal disturbances have not been observed.

#### 3.2.5. Caveats in Individualized Ca and P Supplementation

In individualized mineral supplementation, inorganic Ca or P salts are critical when used for supplementation or as a formula component. Precipitation of Ca/P salts may prevent absorption. For instance, dipotassium phosphate as supplement has been shown to reduce Ca absorption from formula (P source: tribasic calcium phosphate and nonfat dry milk) in a balance study [52]. In a second study, dipotassium phosphate as a formula component prevented the absorption of Ca from Ca-gluconate as a supplement, possibly because precipitation limited the bioavailability of both Ca and inorganic P [55]. On the other hand, organic Ca and P salts have been used successfully (Ca-gluconate and Na_2_-glycerophosphate) [54]. Of note, breast milk phosphates are organically bound to casein micelles, which may be nature’s solution to prevent precipitation [56]. In some units, Ca and P supplements are therefore administered at different times of the day to prevent precipitation. Altogether, supplementation strategies need to be validated [54]. Several additional tools for monitoring and individualizing the adequacy of Ca and P intake have been used or recommended, e.g., serum phosphate, serum alkaline phosphatase, PTH, urinary Ca/Crea and P/Crea quotients, and bone speed of sound. None of them has been prospectively validated so far by providing a management algorithm.

#### 3.2.6. Risks of High Enteral Mineral Supplementation

Sometimes, nurses reported more loose stools with high enteral Ca-gluconate supplementation [54]. On the other hand, stool hardness is associated with stool Ca-soaps and therefore each Ca and P supplement or salt has to be analyzed for efficacy, efficiency and tolerance. Increasing the general Ca and P intake recommendations for all infants may be beneficial for the whole group of preterm infants with regard to bone mineralization; however, it may be harmful for the subgroup of infants with high mineral bioavailability. Urinary excretion of Ca and P may grossly increase in theses infants and increase the incidence of nephrocalcinosis, which has been estimated to occur in at least 7% in VLBW infants [57,58,59,60]. Especially infants with high Ca reabsorption may be affected. Therefore, individually adjusted additional supplementation may be safer. Here the target is to archive a slight Ca and P surplus defined by simultaneous spot urine excretion of both Ca and P at low concentrations.

In summary, previous recommendations for enteral Ca and P intake fail to meet the requirements of preterm infants for two reasons: 1. The average intrauterine Ca and P accretion is systematically underestimated. 2. The average enteral Ca bioavailability is systematically overestimated. The data suggest that promotion of adequate nutrition with sufficient minerals and vitamin D, and possibly an increase in weight-bearing exercise [42] are thus important for former VLBW infants till adulthood [39] and support the hypothesis that improved bone mineralization before discharge may improve lifelong bone health. In hospital, bone mineral accretion largely depends on enteral intake. Therefore, current Ca and P recommendations may be insufficient to meet the needs [2,61]. Higher fortifier/formula mineral content or individual supplementation may be required [54,62].

## 4. Conclusions and Recommendations

### 4.1. Conclusions

1. Ca and P requirements depend on weight gain.
2. Current recommendations for enteral Ca and P intake fail to meet the requirements of preterm infants for two reasons:
  - Given a target neonatal weight gain above 17 g/kg/day, the average intrauterine Ca and P accretion is systematically underestimated.
  - The average enteral Ca bioavailability is systematically overestimated.
3. Promotion of adequate nutrition with sufficient minerals (and vitamin D) may promote long-term bone health.
4. Increasing the enteral intake recommendations for all infants (especially Ca intake) may improve average bone mineralization at the risk of a higher incidence of nephrocalcinosis.

### 4.2. Recommendations

1. Individually adjusted additional enteral mineral supplementation to improve current mineral intake by formula/fortifier should be performed to prevent inadequately low infant BMC and to reduce the risk of nephrocalcinosis.
2. Organic phosphate salts such as glycerophosphate or glucose phosphate should be preferred, because inorganic phosphate salts precipitate much more easily, especially when combined with calcium supplements, thus reducing the bioavailability of both Ca and P.
3. Local individualized Ca and P supplementation strategies should be validated. This includes the idea of separating Ca and P supplement administrations to different times of the day.
4. Future enteral Ca and P recommendation should be based on long-term bone health as well.

### 4.3. Research Direction for Future Studies

The current recommendations for enteral Ca and P intake fail to meet the requirements of preterm infants with regard to long-term bone health. However, it is unclear which level of mineralization must be achieved in preterm infants for prevention of long-term bone mineral deficits. Previous data have shown that there is some degree of bone mineral catch-up in VLBW infants post discharge within the first three years of life [32]. Further studies need to show which degree of reduced bone mineral content may be tolerated at discharge compared to term infants.

## Figures and Tables

**Table 1:** Summary of previous recommendations published since 2000.

	LSRO Klein [5]	Atkinson et al. [1]	ESPGHAN Agostoni et al. [2]	AAP Abrams et al. [3]	Mimouni et al. [4]
Year	2002	2005	2010	2013	2014
Ca mg/kg/day	150–200	120–200	120–140	150–220	120–200
P mg/kg/day	65–90	70–120	60–90	75–140	60–140

## Data Availability

The data presented in this study are available on request from the corresponding author.

## References

[1] Atkinson A.J., Tsang R., Tsang R., Uauy R., Koletzko B., Zlotkin S. (2005). Calcium, magnesium, phosphorus and vitamin D. Nutrition of the Preterm Infant.

[2] Agostoni C., Buonocore G., Carnielli V.P., De Curtis M., Darmaun D., Decsi T., Domellof M., Embleton N.D., Fusch C., Genzel-Boroviczeny O. (2010). Enteral nutrient supply for preterm infants: Commentary from the European Society of Paediatric Gastroenterology, Hepatology and Nutrition Committee on Nutrition. J. Pediatr. Gastroenterol. Nutr..

[3] Abrams S.A., Committee on Nutrition (2013). Calcium and vitamin d requirements of enterally fed preterm infants. Pediatrics.

[4] Mimouni F.B., Mandel D., Lubetzky R., Senterre T. (2014). Calcium, phosphorus, magnesium and vitamin D requirements of the preterm infant. World Rev. Nutr. Diet..

[5] Klein C.J. (2002). Nutrient requirements for preterm infant formulas. J. Nutr..

[6] Hamilton B. (1922). The calcium and phospuorus metabolism of prematurely born infants. Acta Paediatr. Scand..

[7] Oppenheimer S.J., Snodgrass G.J. (1980). Neonatal rickets. Histopathology and quantitative bone changes. Arch. Dis. Child..

[8] Posner A.S. (1969). Crystal chemistry of bone mineral. Physiol. Rev..

[9] Chinoy A., Mughal M.Z., Padidela R. (2019). Metabolic bone disease of prematurity: Causes, recognition, prevention, treatment and long-term consequences. Arch. Dis. Child. Fetal Neonatal Ed..

[10] Rustico S.E., Calabria A.C., Garber S.J. (2014). Metabolic bone disease of prematurity. J. Clin. Transl. Endocrinol..

[11] Faienza M.F., D’Amato E., Natale M.P., Grano M., Chiarito M., Brunetti G., D’Amato G. (2019). Metabolic bone disease of prematurity: Diagnosis and management. Front. Pediatr..

[12] Bozzetti V., Tagliabue P. (2009). Metabolic bone disease in preterm newborn: An update on nutritional issues. Ital. J. Pediatr..

[13] Maas C., Pohlandt F., Mihatsch W.A., Franz A. (2012). Prävention von Knochenmineralmangel bei Frühgeborenen. Klin. Padiatr..

[14] Grahnen H., Sjolin S., Stenstrom A. (1974). Mineralization defects of primary teeth in children born pre-term. Scand. J. Dent. Res..

[15] Pimlott J.F., Howley T.P., Nikiforuk G., Fitzhardinge P.M. (1985). Enamel defects in prematurely born, low birth-weight infants. Pediatr. Dent..

[16] Carroll D.M., Doria A.S., Paul B.S. (2007). Clinical-radiological features of fractures in premature infants—A review. J. Perinat. Med..

[17] Dabezies E.J., Warren P.D. (1997). Fractures in very low birth weight infants with rickets. Clin. Orthop. Relat. Res..

[18] Geggel R.L., Pereira G.R., Spackman T.J. (1978). Fractured ribs: Unusual presentation of rickets in premature infants. J. Pediatr..

[19] Pohlandt F. (1994). Hypothesis: Myopia of prematurity is caused by postnatal bone mineral deficiency. Eur. J. Pediatr..

[20] Pohlandt F. (1994). Bone mineral deficiency as the main factor of dolichocephalic head flattening in very-low-birth-weight infants. Pediatr. Res..

[21] Lyon A.J., McIntosh N., Wheeler K., Williams J.E. (1987). Radiological rickets in extremely low birthweight infants. Pediatr. Radiol..

[22] Koo W.W., Sherman R., Succop P., Krug-Wispe S., Tsang R.C., Steichen J.J., Crawford A.H., Oestreich A.E. (1989). Fractures and rickets in very low birth weight infants: Conservative management and outcome. J. Pediatr. Orthop..

[23] Fewtrell M.S. (2011). Does early nutrition program later bone health in preterm infants?. Am. J. Clin. Nutr..

[24] Viswanathan S., Khasawneh W., McNelis K., Dykstra C., Amstadt R., Super D.M., Groh-Wargo S., Kumar D. (2014). Metabolic bone disease: A continued challenge in extremely low birth weight infants. J. Parenter. Enteral. Nutr..

[25] Smurthwaite D., Mughal M.Z., Wright N.B., Russell S., Emmerson A.J. (2009). How common are radiologically apparent rib fractures in extremely low birth weight preterm infants?. Arch. Dis. Child..

[26] Mitchell S.M., Rogers S.P., Hicks P.D., Hawthorne K.M., Parker B.R., Abrams S.A. (2009). High frequencies of elevated alkaline phosphatase activity and rickets exist in extremely low birth weight infants despite current nutritional support. BMC Pediatr..

[27] Abdallah E.A., Said R.N., Mosallam D.S., Moawad E.M., Kamal N.M., Fathallah M.G. (2016). Serial serum alkaline phosphatase as an early biomarker for osteopenia of prematurity. Medicine.

[28] Lapillonne A.A., Glorieux F.H., Salle B.L., Braillon P.M., Chambon M., Rigo J., Putet G., Senterre J. (1994). Mineral balance and whole body bone mineral content in very low-birth-weight infants. Acta Paediatr..

[29] Wauben I.P., Atkinson S.A., Grad T.L., Shah J.K., Paes B. (1998). Moderate nutrient supplementation of mother’s milk for preterm infants supports adequate bone mass and short-term growth: A randomized, controlled trial. Am. J. Clin. Nutr..

[30] Pieltain C., de Halleux V., Senterre T., Rigo J. (2013). Prematurity and bone health. World Rev. Nutr. Diet..

[31] Bergner E.M., Shypailo R., Visuthranukul C., Hagan J., O’Donnell A.R., Hawthorne K.M., Abrams S.A., Hair A.B. (2020). Growth, body composition, and neurodevelopmental outcomes at 2 years among preterm infants fed an exclusive human milk diet in the neonatal intensive care unit: A pilot study. Breastfeed. Med..

[32] Mihatsch W., Dorronsoro Martín I., Barrios-Sabador V., Couce M.L., Martos-Moreno G.Á., Argente J., Quero J., Saenz de Pipaon M. (2021). Bone mineral density, body composition, and metabolic health of very low birth weight infants fed in hospital following current macronutrient recommendations during the first 3 years of life. Nutrients.

[33] Fewtrell M.S., Prentice A., Jones S.C., Bishop N.J., Stirling D., Buffenstein R., Lunt M., Cole T.J., Lucas A. (1999). Bone mineralization and turnover in preterm infants at 8–12 years of age: The effect of early diet. J. Bone Miner. Res..

[34] Abou Samra H., Stevens D., Binkley T., Specker B. (2009). Determinants of bone mass and size in 7-year-old former term, late-preterm, and preterm boys. Osteoporos. Int..

[35] Zamora S.A., Belli D.C., Rizzoli R., Slosman D.O., Bonjour J.P. (2001). Lower femoral neck bone mineral density in prepubertal former preterm girls. Bone.

[36] Martinez-Mesa J., Restrepo-Mendez M.C., Gonzalez D.A., Wehrmeister F.C., Horta B.L., Domingues M.R., Menezes A.M. (2013). Life-course evidence of birth weight effects on bone mass: Systematic review and meta-analysis. Osteoporos. Int..

[37] Balasuriya C.N.D., Evensen K.A.I., Mosti M.P., Brubakk A.M., Jacobsen G.W., Indredavik M.S., Schei B., Stunes A.K., Syversen U. (2017). Peak bone mass and bone microarchitecture in adults born with low birth weight preterm or at term: A cohort study. J. Clin. Endocrinol. Metab..

[38] Buttazzoni C., Rosengren B., Tveit M., Landin L., Nilsson J.A., Karlsson M. (2016). Preterm children born small for gestational age are at risk for low adult bone mass. Calcif. Tissue Int..

[39] Hovi P., Andersson S., Jarvenpaa A.L., Eriksson J.G., Strang-Karlsson S., Kajantie E., Makitie O. (2009). Decreased bone mineral density in adults born with very low birth weight: A cohort study. PLoS Med..

[40] Xie L.F., Alos N., Cloutier A., Beland C., Dubois J., Nuyt A.M., Luu T.M. (2019). The long-term impact of very preterm birth on adult bone mineral density. Bone Rep..

[41] Haikerwal A., Doyle L.W., Patton G., Garland S.M., Cheung M.M., Wark J.D., Cheong J.L.Y. (2021). Bone health in young adult survivors born extremely preterm or extremely low birthweight in the post surfactant era. Bone.

[42] Engan M., Vollsaeter M., Oymar K., Markestad T., Eide G.E., Halvorsen T., Juliusson P., Clemm H. (2019). Comparison of physical activity and body composition in a cohort of children born extremely preterm or with extremely low birth weight to matched term-born controls: A follow-up study. BMJ Paediatr. Open.

[43] Fee B.A., Weil W.B. (1963). Body composition of infants of diabetic mothers by direct analysis. Ann. N. Y. Acad. Sci..

[44] Kelly H.J., Soloan R.E., Hoffman W., Saunders C. (1951). Accumulation of nitrogen and six minerals in the human fetus during gestation. Hum. Biol..

[45] Widdowson E.M., Dickerson J.W.T., Comar C.L., Bronner F. (1964). Chemical compostion of the body. Mineral Metabolism.

[46] Ellis K.J., Shypailo R.J., Schanler R.J. (1994). Body composition of the preterm infant. Ann. Hum. Biol..

[47] Rigo J., De Curtis M., Picaud J.C., Nyamugabo K., Senterre J. (1998). Whole body calcium content in term and preterm neonates. Eur. J. Pediatr..

[48] Voigt M., Schneider K.T., Jährig K. (1996). Analysis of a 1992 birth sample in Germany. 1: New percentile values of the body weight of newborn infants. Geburtsh. Frauenheilkd..

[49] Alexander G.R., Himes J.H., Kaufman R.B., Mor J., Kogan M. (1996). A United States National reference for fetal growth. Obstet. Gynecol..

[50] Hay W.W., Caballero B. (2021). Growth and development: Physiological aspects. Encyclopedia of Human Nutrition.

[51] Bronner F., Salle B.L., Putet G., Rigo J., Senterre J. (1992). Net calcium absorption in premature infants: Results of 103 metabolic balance studies. Am. J. Clin. Nutr..

[52] Mize C.E., Uauy R., Waidelich D., Neylan M.J., Jacobs J. (1995). Effect of phosphorus supply on mineral balance at high calcium intakes in very low birth weight infants. Am. J. Clin. Nutr..

[53] Maas C., Pohlandt F., Mihatsch W.A., Franz A.R. (2012). Prevention of bone mineral deficiency in premature infants: Review of the literature with focus on monitoring of urinary calcium and phosphate. Klin. Padiatr..

[54] Trotter A., Pohlandt F. (2002). Calcium and phosphorus retention in extremely preterm infants supplemented individually. Acta Paediatr..

[55] Carroll W.F., Fabres J., Nagy T.R., Frazier M., Roane C., Pohlandt F., Carlo W.A., Thome U.H. (2011). Results of extremely-low-birth-weight infants randomized to receive extra enteral calcium supply. J. Pediatr. Gastroenterol. Nutr..

[56] Atkinson S.A., Alston-Mills B., Lonnerdal B., Neville M.C., Jensen R.G. (1995). Major minerals and ionic constituents of human and bovine milks. Handbook of Milk Composition.

[57] Narendra A., White M.P., Rolton H.A., Alloub Z.I., Wilkinson G., McColl J.H., Beattie J. (2001). Nephrocalcinosis in preterm babies. Arch. Dis. Child. Fetal Neonatal Ed..

[58] Hein G., Richter D., Manz F., Weitzel D., Kalhoff H. (2004). Development of nephrocalcinosis in very low birth weight infants. Pediatr. Nephrol..

[59] Schell-Feith E.A., Kist-van Holthe J.E., van der Heijden A.J. (2010). Nephrocalcinosis in preterm neonates. Pediatr. Nephrol..

[60] Hoppe B., Duran I., Martin A., Kribs A., Benz-Bohm G., Michalk D.V., Roth B. (2002). Nephrocalcinosis in preterm infants: A single center experience. Pediatr. Nephrol..

[61] Mihatsch W.A., Braegger C., Bronsky J., Cai W., Campoy C., Carnielli V., Darmaun D., Desci T., Domellof M., Embleton N. (2018). ESPGHAN/ESPEN/ESPR/CSPEN guidelines on pediatric parenteral nutrition. Clin. Nutr..

[62] Pohlandt F. (1994). Prevention of postnatal bone demineralization in very low-birth-weight infants by individually monitored supplementation with calcium and phosphorus. Pediatr. Res..

